# Alexis O-ring wound retractor versus traditional metal retractors for the prevention of postcaesarean surgical site infections

**DOI:** 10.4102/safp.v65i1.5651

**Published:** 2023-02-16

**Authors:** Marabe S. Mothiba, Tshimane C. Tshepuwane, Adegoke O. Adefolalu, Tshweu S. Monokoane

**Affiliations:** 1 Department of Obstetrics and Gynaecology, Sefako Makgatho Health Sciences University, Pretoria, South Africa; 2 Practice of Medicine Integrated Programme, School of Medicine, Sefako Makgatho Health Sciences University, Pretoria, South Africa

**Keywords:** metal retractors, wound retraction, Alexis^®^ O-ring retractors, plastic sheath wound retractor, surgical site infection, caesarean section

## Abstract

**Background:**

Postcaesarean surgical site infections (SSI) remain a burden globally. The Alexis^®^ O C-Section Retractor, a plastic sheath retractor known to have decreased incidence of SSIs in gastrointestinal surgery, is yet to have its efficacy established during caesarean section (CS). This study aimed to compare the differences in the rate of postcaesarean surgical wound site infections between the Alexis^®^ retractor and traditional metal retractors during CS at a large tertiary hospital in Pretoria.

**Methods:**

Pregnant women scheduled for elective CS were prospectively randomised to either the Alexis^®^ retractor group or the traditional metal retractor group at a tertiary hospital in Pretoria between August 2015 and July 2016. The defined primary outcome was development of SSI, and secondary outcomes comprised patients’ peri-operative parameters. All participants’ wound sites were observed in the hospital for 3 days before discharge and again at 30 days postpartum. Data were analysed using SPSS version 25 with *p* < 0.05 considered significant.

**Results:**

A total of 207 participants were involved, Alexis^®^ (*n* = 102) and metal retractors (*n* = 105). None of the participants developed postsurgical site wound infection after 30 days, and there were no differences in time to delivery, total operative time, estimated blood loss or postoperative pain between the two arms of study.

**Conclusion:**

The study found no difference in participants’ outcomes using the Alexis^®^ retractor in comparison with the traditional metal wound retractors. We suggest that the use of Alexis^®^ retractor be at the surgeon’s discretion and its routine use not advised for now.

**Contribution:**

This research being the first of its kind in South Africa in which patients’ clinical outcomes were compared post caesarean section from Alexis’s plastic sheath group and metal retractors group in an attempt to proffer solution to the high burden of SSI. Although no difference was seen at this point, the research was pragmatic, as it was carried out in a setting with high burden of SSI. The study is going to serve as a baseline against which studies carried out in future can be compared.

## Introduction

Caesarean section (CS) is among the most frequently performed major surgical operations, being a critical life-saving procedure, but the annual rate at which it is performed continues to increase exponentially to the extent that it now accounts for almost 20% of all births globally.^1,2,3^ Its usefulness can never be overemphasised in the context of obstetrics practice; however, unlike uncomplicated vaginal birth, CS is associated with quite a few complications, most especially postsurgical site infection (SSIs). The association between increased CS rate and postcaesarean SSIs has been described in the literature. Surgical site infections were found to be the commonest postoperative complication following CS, and they ranked third on the list of hospital-acquired infections.^4,5^ In addition, nearly 10% of all CSs have SSIs as sequelae, and the risk of SSIs increases about five times following CS compared with vaginal birth.^4,5,6^ Wound sepsis contributes significantly to maternal morbidity and mortality, especially when other aggravating factors such as prolonged labour, prolonged rupture of membranes and long duration of operation are present.^6^ The incidence rate of SSIs widely varies, as shown by different authors, and could depend on factors like hospital settings, surgeons’ skills, availability of resources and geographical locations. Overall, the incidence rate of SSIs can be as low as 3%, and up to 14% in some places, depending on the prevailing circumstances.^7,8,9^

There has been some improvement in terms of innovations and strategies directed at infection control measures to reduce the incidence of SSI in settings where it remains very high. Some of these include identification of factors known to influence postcaesarean SSIs such as obesity, previous CS, human immunodeficiency virus (HIV), acquired immune deficiency syndrome (AIDS), immunosuppressive disorders, chorioamnionitis, steroid treatment, use of staple suture wound closure, emergency CS, excessive blood loss and nonadherence to prescribed wound care management after leaving the hospital.^10,11^ Furthermore, measures such as improved operating room ventilation, periodic training of surgeons, adoption of modern surgical techniques, advanced sterilisation practices, use of barriers during operation and antimicrobial prophylaxis when indicated are some of the modalities employed to reduce post-CS infections. Despite all the above, the incidence rate of SSIs remains at unacceptable levels, especially in the low- and middle-income countries (LMIC), where it is among the commonest causes of morbidity and mortality.^7,8^ In several of these LMICs, CS is classified as essential among surgical procedures recognised as important components of public healthcare services; this means it is an essential service that must be always be made available. Given the resource-constrained conditions prevalent in most of these LMICs, their higher CS volume seemed to have worsened the incidence rate of postoperative SSIs.^1^

Despite having decent healthcare coverage and substantial advances in postnatal care, puerperal sepsis and SSI remain among the leading causes of maternal morbidity and mortality in South Africa. Post-CS SSIs and their associated factors have been previously reported in South Africa; a lack of skilled personnel, environmental risk factors and some other personal factors were all described as enablers of SSIs.^7,11,12^ The situation at the study setting, a large maternity unit of a tertiary hospital in Pretoria with an annual CS rate of between 30% and 40%, and a postcaesarean infection rate of nearly 10%, is not too different from what has been described above. Therefore, the need for continuous innovations aimed at reducing incidence of postcaesarean SSIs is relevant, most especially in the study setting as a lower- and middle-income countries, a part of LMICs, with constrained resources. Traditionally, metal wound retractors are usually used during CS; however, the advent of newer retractors such as the Alexis^®^ O C-Section Retractor, a single-use device made of a flexible polymer membrane formed into a cylindrical shape, serves a dual purpose as a wound retractor and protector.^13^ The device has been used to provide adequate and atraumatic wound retraction during surgery, together with its ability to reduce postsurgical infections, particularly during gastrointestinal surgeries.^13,14,15,16,17,18,19^ The environmental impact of producing the Alexis^®^ retractors has to be balanced with the benefits derived from its clinical use in the long term. In comparison with traditional metal retractors, the relatively high cost of the Alexis^®^ retractors prohibits their routine use currently.^5,6,16^ Researchers opined that if Alexis^®^ retractors show significant SSI reduction during CS, the high cost would be justified considering the overall impact of SSI on the healthcare system and patients.^16^ Surgical site infections have been reported to increase the cost of healthcare by nearly $3 – $10 billion because of the number of patients who develop SSI and the resultant high risk of death, which often complicates SSI. Furthermore, on average, most SSI patients spend an extra 7–11 days in hospital, and about 60% of them will eventually end up in the intensive care unit. In terms of being readmitted to hospital, SSI patients are five times more likely to be readmitted compared to patients without SSI.^5,6,7^

The Alexis^®^ retractor has been previously used in the study setting during CS as a wound retractor and protector. Based on anecdotal evidence, the retractor appeared to have positively influenced some patients’ peri-operative outcomes. However, there is insufficient evidence from the few studies that have assessed its efficacy and comparative advantage over the traditional metal retractors for one to advocate for its routine use. Therefore, the current study was designed to compare the peri-operative outcomes among pregnant women undergoing caesarean delivery using the Alexis^®^ retractor with a similar cohort of pregnant women for whom metal wound retractors were used. The primary aim was to determine whether the Alexis^®^ retractor reduces the risk of SSI in comparison with metal wound retractors.

## Method

### Study design and setting

The research design for the study employed a quantitative method in its approach, using a double-blinded, prospective, randomised controlled study that was carried out among a target population of pregnant women scheduled for CS at the maternity unit.

### Sampling and study population

Sample size calculation was based on the SSI rate comparison between Alexis^®^ retractor and the conventional metal wound retractors. Given that the SSI incidence rates in the metal retractor group and Alexis^®^ retractor group were 10% (average SSI incidence with traditional retractors) and 1% (anticipated incidence), respectively, the sample size required to observe this difference with a study power of 80% was 100 participants in each arm. Statistical Package for the Social Sciences (SPSS) for Windows (version 25). Therefore, the sample size was 200 participants in total, with seven additional patients making it 207 to allow for possible attrition or withdrawal, with the Alexis^®^ group having 102 participants and the other 105 participants.

The sampled participants and the principal investigator (first author) were blinded to the patients’ group allocation and the type of retractor used during the individual surgery to ensure scientific rigour. The *exclusion criteria* included pregnant women younger than 18 years and older than 34 years of age, anticipated complicated delivery, multiparous pregnancy, patients with previous CS and cases of suspected infection. The following *inclusion criteria* were: 18–34 years of age, and pregnancy in the third trimester. The participants were randomly allocated on an alternate basis to the two study groups, namely the Alexis^®^ retractor group and the control group (other metal retractors), as they presented for surgery after being assessed for eligibility by an assistant, who was a surgeon in the maternity unit and was not involved in the study. The decision regarding the necessity of the CS in every one of the participants was initially made by the attending doctor (registrar) in consultation with the supervising consultant. This decision was further reviewed and ratified by a second consultant after independently verifying the need for the procedure in the patient. Overall, at least two senior consultants corroborated the decision to perform CS on all pregnant women recruited before they were eventually entered as study participants.

### Data collection

Over the 12-month study period, a total of 207 pregnant women were recruited for the study; the study group of patients (Alexis^®^ retractor) comprised 102 women and the control group (metal retractor) had 105 participants. All the participants were thoroughly briefed about the purpose of the research, and they voluntarily gave written informed consent to participate in the study. The risks for all study participants were not increased beyond that which exists for a CS. All the surgical operations were carried out by competent surgeons who were trained on how to use the Alexis^®^ retractor before the start of the study in the maternity unit at the hospital, adhering to laid-down protocols for performing a CS.

### Data analysis

The data were analysed with IBM^®^ Statistical Package for the Social Sciences (SPSS) Statistics 25 (IBM Corporation, Armonk, New York, United States), and the peri-operative outcomes were summarised descriptively and compared between the two study groups using *t*-tests. Statistical significance testing was two-sided at the 0.05 level. The peri-operative outcomes from the surgical procedures were compared between the two groups using the following parameters immediately after surgery and throughout admission postoperatively: drugs used in the ward, estimated blood loss, postoperative temperature and pulse, soiling of garments, wound hematoma and serous ooze from the wound. All study subjects were asked to complete a patient assessment of pain and comfort, using a numerical rating scale, and the scores were charted each time observation was carried out. The participants were observed in the hospital ward for 3 days after the CS, using a checklist prepared for that purpose by the attending surgeon who completed the assessment form for the above parameters. At the point of discharge from the hospital, the participants were given sealed forms which contained the parameters needed to evaluate their postoperative outcomes, and the forms were to be completed by the attending doctor, should there be any need for such a participant to consult a doctor at any time before their first scheduled visit at the hospital (30 days post operation). The following parameters were included in the discharge form: postoperative pain, postoperative temperature and pulse, soiling of garments, wound hematoma, serous ooze from the wound and infection of the wound at postnatal check-ups (i.e. SSI). In this study, SSI was operationally defined as ‘any infection of the superficial or deep tissues or the organ/space affected by the surgery, and which occurs within 30 days of surgery’,^4^ and it was expected that such SSI would be confirmed by an independent observer, a senior specialist obstetrician and gynaecologist in the hospital who was not involved in the research at all.

### Ethical considerations

Ethical approval was obtained from the institution’s Research and Ethics Committee (ref. no. SMUREC/M/124/2015: PG), and permission to carry out the study was granted by the hospital management.

## Results

A total of 207 participants were involved in this research, 102 in the Alexis^®^ group and 105 in the metal retractors group, with no single attrition recorded. The results of the study are highlighted in Table 1. The baseline characteristics were similar between the two groups, with a median age of 26 years for both groups. In both groups, the operating time for most of the participants ranged between 31 min and 60 min. In the Alexis^®^ arm, the majority of the procedures (51%) lasted between 31 min and 60 min; this was slightly higher in the metal retractor group, where the majority (54.3%) of all the procedures also lasted between 31 min and 60 min. The mean duration of the procedure was 54.65 min (standard deviation [s.d.] = 25.5) for the Alexis^®^ and 56.22 min (s.d. = 28.0) for the metal retractor (*p* = 0.78), which showed there is no statistical difference in duration of surgery between the two groups. As shown in Table 2, in terms of blood loss, the amount of blood loss was minimal in both study groups, as only two participants each from both groups lost more than 1000 mL of blood during the surgical operations. This accounts for less than 2% for each arm when compared together with a *p*-value of 0.129, indicating no difference in the volume of blood lost between participants in the Alexis^®^ and the metal retractor groups. The pulse rates recorded for the participants during the 3-day observation period were within normal range, mostly falling between 50 and 100 beats per minute for both the Alexis^®^ and metal retractor arms. Meanwhile, the mean pulse rates for the observations made from Day 1, Day 2 and Day 3 were 76.05 (s.d. = ± 13.89), 75.25 (s.d. = ± 12.78) and 74.94 (s.d. = ± 12.20), respectively, for the Alexis^®^ group. The mean pulse rates of the metal retractor group were 82.50 (s.d. = ± 11.33), 82.73 (s.d. = ± 11.85) and 83.19 (s.d. = ± 11.02) on Day 1, Day 2 and Day 3, respectively, with a *p*-value of 0.0001, indicating a significant difference in the pulse rates observed between the participants in two groups, as depicted in Table 2.

**TABLE 1:** Measurement of outcome parameters between the two groups (*n* = 207).

Characteristics	Alexis^®^ (*n* = 102)		Metal retractors (*n* = 105)		Total	%
	Freq	%	Freq	%		
**Duration of surgery**						
15–30 min	15	14.7	18	17.1	33	16.0
31–60 min	52	51.0	57	54.3	109	52.7
61–120 min	34	33.4	28	26.7	62	29.9
121–180 min	1	0.9	2	1.9	3	1.4
**Type of anaesthesia**						
General	1	0.98	6	5.7	7	3.4
Spinal	101	99.01	99	94.3	200	96.6
**Postoperative pain**						
No	102	100.0	105	100.0	207	100.0
Yes	0	0.0	0	0.0	0	0.0
**Blood loss**						
< 1000 mL	100	98.04	103	98.1	203	98.0
> 1000 mL	2	1.96	2	1.9	4	2.0
**Postoperative fever**						
No	102	100.0	105	100.0	207	100.0
Yes	0	0.0	0	0.0	0	0.0
**Postoperative serous ooze**						
No	102	100.0	105	100.0	207	100.0
Yes	0	0.0	0	0.0	0	0.0
**Postoperative garment soiling**						
No	102	100.0	105	100.0	207	100.0
Yes	0	0.0	0	0.0	0	0.0
**Postoperative wound haematoma**						
No	102	100.0	105	100.0	207	100.0
Yes	0	0.0	0	0.0	0	0.0

Freq, frequency.

**TABLE 2:** Comparison of outcome parameters between the two groups (*n* = 207).

Parameters	Alexis^®^ (*n* =102)		Metal retractor (*n* = 105)		*p*
	Mean	s.d.	Mean	s.d.	
Duration of surgery (min)	54.65	± 25.46	56.22	± 28.02	0.787
Blood loss (mL)	541.47	± 196.32	589.14	± 249.93	0.129
Pulse rate (bpm)	75.415	± 12.61	82.81	± 10.27	< 0.001

s.d., standard deviation; bpm, beats per minute.

The type of anaesthesia which was mostly used was spinal anaesthesia; it was used 101 times in the Alexis^®^ group and 99 times in the other group, accounting for 96.6% of the total, and on very few instances was general anaesthesia administered in either group, as shown in Table 1.

For both groups, there was no postoperative pain, no soiling of garments, no wound hematoma and no serous ooze, and no antibiotics were needed. Wound closure was evident in both. The body temperature recorded was normal for all the participants in both groups over the 3 days. When the participants were assessed for pain, they mostly mentioned that they experienced minor discomfort at the incision site, which was not unusual for the kind of procedure they underwent. Pethidine was the standard analgesia used across the board for both groups when indicated. In addition, there were no cases of nausea, excruciating pain, fever, pus or malodorous smell reported by the participants or observed during the research. Therefore, one can safely conclude that out of the 207 participants, in which the Alexis^®^ group had 102 patients and the metal wound retractor had 105 patients, no patient developed surgical site wound infection.

## Discussion

Postcaesarean SSIs appear to be more prevalent than has previously been reported, as they top the list of reasons why patients’ hospital stays are sometimes prolonged.^5^ A normal surgical incision heals between 4 and 6 weeks if there is no peri-operative factor that affects wound healing.^19^ In terms of pathogenesis, postpartum infections often originate from an endogenous source such as the maternal body flora reaching the endometrium or cervix, but they could also be from an exogenous source, where contamination from the environment leads to wound infection and eventually sepsis, a phenomenon that is far too common in obstetrics practice.^19^ The various wound protection methods used during obstetric surgeries were designed such that they provide adequate exposure and minimal trauma to the wound edges, thereby reducing the chances of postsurgical wound infection. Recent studies have shown that specialised plastic sheath retractors seem more effective than the traditional metal retractors in terms of preventing postsurgical wound infection.^13^ One such study revealed a significant reduction of surgical site wound infection with plastic sheath retractors in comparison with traditional metal retractors, in which SSI was eight times more likely to occur compared to the Alexis^®^ retractor.^13^ This study had 100 patients allocated in the metal wound retractor group and 98 patients allocated in the Alexis^®^ wound retractor arm; the present study with the same number of patients did not show any difference as there were no patients with sepsis from either group, indicating no comparative advantage between Alexis^®^ and metal retractors. This is in keeping with the findings of other studies that found no difference in SSIs with the Alexis^®^ retractor and traditional metal retractors. One of these is a study conducted in the United States that confirmed there was no significant difference in SSIs with the use of the Alexis^®^ in women undergoing caesarean delivery at term.^14^ The study included 536 patients and did not find any difference in time to delivery, total operative time, estimated blood loss or postoperative pain.^14^ In another study conducted among obese pregnant women undergoing CS, the use of the Alexis^®^ retractor did not decrease the incidence of postcaesarean SSI among a population of obese pregnant women.^15^ Furthermore, in another prospective study of more than 230 women in which the Alexis^®^ plastic sheath retractor and Doyen’s retractor were compared together, no evidence of SSI was reported, as defined in terms of wound dehiscence, pain or tenderness in the lower abdomen, localised swelling, redness, heat or purulent discharge from the wound in any of the participants.^17^ Although an incidental finding of a significant difference in some participants’ pulse rate over 3 days after CS was seen in the present study’s results, this remains an isolated finding which cannot be used as a significant marker for sepsis. None of the patients eventually developed sepsis on follow-up at 30 days after CS. Therefore, the findings above as revealed in earlier studies have been corroborated by this study’s results, as no significant difference was found in the study participants when comparing the Alexis^®^ retractor with the traditional metal wound retractor.

Despite the lack of convincing evidence of the Alexis^®^ wound retractor’s benefit during CS, there have been many instances during abdominal surgery in which the use of the Alexis^®^ retractor showed significant benefit in preventing SSI. Various studies in which the Alexis^®^ retractor was compared with metal retractors during gastrointestinal surgery showed that Alexis^®^ wound retractor was more effective in preventing SSI.^18,20,21,22^ Nevertheless, this apparent usefulness of Alexis^®^ retractors during abdominal surgery was not consistent across wound assessments performed by clinicians and those reported by patients post laparotomy, where it was asserted that wound edge protection devices do not reduce the rate of SSI at all.^23^

## Conclusion

In conclusion, if one takes into consideration all the available evidence after comparing the Alexis^®^ with the traditional metal retractors, it is difficult to overlook the pockets of success recorded among small subgroups in which the Alexis^®^ retractors appeared useful. Other than that, it is beginning to emerge that Alexis^®^ retractors do not influence the incidence of postcaesarean SSIs in the study setting. As healthcare interventions become very expensive as a result of advancements in medical technology, it is crucial to ensure that all surgical interventions employed at any point are measured up with tangible benefits to the patients. Therefore, considering the above-mentioned facts and its high cost, the routine use of the Alexis^®^ retractor during CS is not supported by available evidence. Its use as a retractor should be left to the discretion of the surgeon and clinical circumstances. Finally, further research is still needed on this topic, and future studies should attempt to identify the patient population groups in which these retractors could be of great benefit.
